# MicroRNA-145-5p and microRNA-320a encapsulated in endothelial microparticles contribute to the progression of vasculitis in acute Kawasaki Disease

**DOI:** 10.1038/s41598-018-19310-4

**Published:** 2018-01-17

**Authors:** Hideyuki Nakaoka, Keiichi Hirono, Seiji Yamamoto, Ichiro Takasaki, Kei Takahashi, Koshi Kinoshita, Asami Takasaki, Naonori Nishida, Mako Okabe, Wang Ce, Nariaki Miyao, Kazuyoshi Saito, Keijiro Ibuki, Sayaka Ozawa, Yuichi Adachi, Fukiko Ichida

**Affiliations:** 1 Department of Pediatrics, Faculty of Medicine, University of Toyama, Toyama, Japan; 2 Department of Pathology, Faculty of Medicine, University of Toyama, Toyama, Japan; 3 Division of Molecular Genetics Research, Life Science Research Center, University of Toyama, Toyama, Japan; 4 Department of Pathology, Toho University Ohashi Medical Center, Tokyo, Japan; 5 Department of Legal Medicine, Faculty of Medicine, University of Toyama, Toyama, Japan

## Abstract

Kawasaki Disease (KD) is an acute inflammatory disease that takes the form of systemic vasculitis. Endothelial microparticles (EMPs) have been recognized as an important transcellular delivery system. We hypothesized whether EMPs are involved in vasculitis in acute KD. Fifty patients with acute KD were enrolled, divided into two subgroups: those with coronary artery lesions (CAL) (n = 5) and those without CAL (NCAL) (n = 45). EMPs were measured using flow cytometry, and microRNA (miR) expression profiling was performed by microRNA array. The percentage of EMPs in acute KD was significantly higher than in controls (*P* < 0.0001). EMPs in patients with CAL rapidly increased after the initial treatment, and was significantly higher than those in NCAL (*P* < 0.001). In patients with CAL, we identified 2 specific miRs encapsulated in EMPs, hsa-miR-145-5p and hsa-miR-320a, which are predicted to affect monocyte function using *in silico* analysis, and were demonstrated to upregulate inflammatory cytokine mRNAs in THP-1 monocytes. *In situ* hybridization confirmed that hsa-miR-145-5p was preferentially expressed in CAL. EMPs may serve as a sensitive marker for the severity of vasculitis in acute KD. Moreover, these 2 specific miRs encapsulated in EMPs might be involved in inflammatory cytokine regulation and the pathogenesis of vasculitis in acute KD.

## Introduction

Kawasaki Disease (KD) is typically an acute inflammatory syndrome that takes the form of systemic vasculitis^1^. Although many studies have suggested that infectious agents are involved in the pathogenesis of KD^2^, the etiology of the disease remains largely unknown. The acute inflammation and subsequent reparative process may lead to irreversible changes in arterial structure^3,4^. Although, administration of high-dose intravenous immunoglobulin (IVIG) during the acute phase of KD can reduce the occurrence of coronary artery lesions (CAL), destruction of arterial structures still continue, especially in non-responders^3,4^.

To date, many studies have reported that the release of particles from activated or apoptotic cells, and demonstrated that such particles are commonly found in human plasma as microparticles (MPs)^5,6^. MPs usually refer to membranous particles between 100 nm and 1.0 μm in diameter. MPs are considered to be both biomarkers and effectors of cell signaling that maintain and/or initiate cell dysfunction^6^. Further, where inflammation occurs on the vascular endothelial cells, endothelial microparticles (EMPs), a particular fraction of MPs in circulating blood, are released from the cellular surface of endothelial cells^5,7^.

Recent studies have demonstrated that endothelial dysfunction is also a predictor of future coronary events and the disease state of the coronary artery^8^, and that endothelial cell dysfunction may be detected clinically by measuring the impairment of endothelium-dependent vasodilation^9^. EMPs are often observed in circulating blood as small-sized membranous particle-like soluble factors. EMPs are produced from activated endothelial cells by many kinds of environmental stresses, and their composition can be used to characterize the status of the endothelial cells^7^. EMPs are now appreciated as an important transcellular delivery system in the exchange of biological signals, and typically contain microRNAs (miRs)^7,10^. MiRs are non-coding, single stranded RNAs, 18–24-nucleotide in length, that exert the ability to negatively regulate the expression of target genes, and are involved in several cellular processes including cell proliferation, apoptosis, migration, invasion, and stress response^11–13^. In addition, EMPs can transduce cellular signals from the endothelial cells to various target cells by encapsulated small molecules^7^, or alternatively through secretion of soluble mediators and effectors^14^.

To date, there have been few studies investigating the role of miRs that can be encapsulated in EMPs, in patients with acute KD. It is still unknown whether these miRs can communicate with various target cells, and regulate inflammatory cytokines. In this study, we hypothesized that EMPs could serve as a sensitive marker of vasculitis in acute KD, and contain bioactive molecules. Our findings suggest that specifically identified miRs may be encapsulated in EMPs and can modulate cytokine expression levels in recipient cells.

## Results

### Clinical characteristics

Demographic data of each group show that there were no significant differences between the patients with KD and the controls, except for CRP levels, white blood cell counts, platelet counts and duration of fever, which were tended to higher/longer in the KD patients (Table 1). In the KD patients with CAL, the duration of fever was longer than those with NCAL. All of the patients with CAL were refractory to IVIG treatment. Other parameters were not significantly different between the 2 groups (Table 2).

**Table 1:** Demographic and clinical characteristics of enrolled patients.

	Kawasaki Disease (n = 50)	Non-KD Febrile (n = 25)	Healthy (n = 25)
Age, month, mean (range)	37 (4–169)	44 (4–140)	49 (3–146)
Male sex, n (%)	30 (60.0)	12 (48.0)	12 (48.0)
Refractory, n (%)	13 (26.0)	−	−
Z score of CA > 2.5, n (%)	5 (10.0)	—	—
CRP, mg/dl, mean ± SD	8.5 ± 0.6^*^	3.4 ± 1.0	0.1 ± 0.0
White blood cell count, /mm^3^, mean ± SD	16,667 ± 809^*^	11,596 ± 950	7,805 ± 425
Neutrophils, %, mean ± SD	72.0 ± 1.8	65.3 ± 3.9	42.3 ± 1.5
Platelet count, ×10^4^/mm^3^, mean ± SD	35.0 ± 1.3^*^	23.8 ± 1.5	27.3 ± 1.5
Na, mmol/l, mean ± SD	134.4 ± 0.4	135.2 ± 0.9	139.1 ± 0.4
AST, IU/L, mean ± SD	160.0 ± 48.5	38.0 ± 2.9	34.9 ± 3.6
Duration of fever, days, mean ± SD	7.8 ± 0.4^*^	3.1 ± 0.3	—

Demographic data of KD patients and two control groups. There were no significant differences between the KD patients and the control groups, except for CRP levels, white blood cell counts, platelet count and duration of fever which were higher in the KD patients. *P < 0.05.

**Table 2:** Demographic data of KD patients.

	Kawasaki Disease (n = 50)	
	KD with CAL (n = 5)	KD without CAL (n = 45)
Age, month, mean (range)	39 (4-44)	17 (9-169)
Male sex, n (%)	4 (80.0)	26 (57.8)
Refractory, n (%)	4 (80.0)	9 (20.0)
CRP, mg/dl, mean ± SD	10.5 ± 2.2	8.3 ± 0.6
White blood cell count, /mm^3^, mean ± SD	20, 976 ± 2,661	16,188 ± 828
Neutrophils, %, mean ± SD	73.7 ± 5.5	71.8 ± 2.0
Platelet count, ×10^4^/mm^3^, mean ± SD	40.3 ± 4.2	34.4 ± 1.3
Na, mmol/l, mean ± SD	135.8 ± 1.5	134.2 ± 0.4
AST, IU/L, mean ± SD	230.4 ± 201.2	152.2 ± 49.9
Duration of fever, days, mean ± SD	12.5 ± 2.6*	7.3 ± 0.4

In the KD patients with CAL the duration of fever was higher than those without CAL (*P < 0.05). Other parameters were not significantly different between the 2 groups.

### Relationships between EMPs and CAL

**Figure 1:**
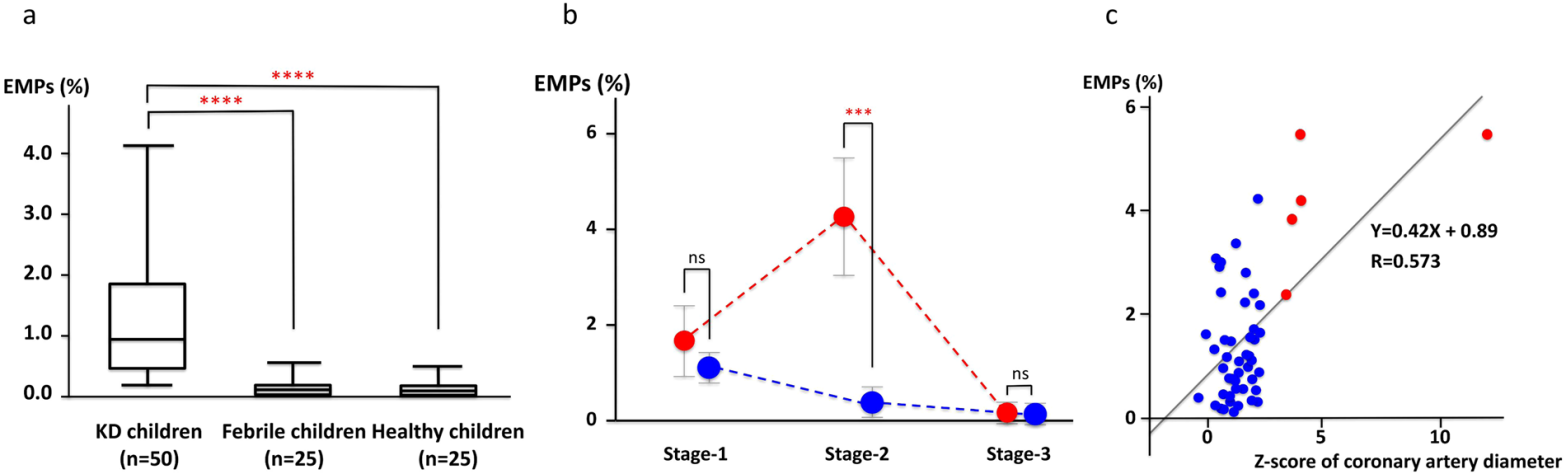
Relationships between EMPs and the KD patients with or without CAL. (**a**) EMPs are significantly upregulated in patients with KD among the enrolled patients. The percentage of EMPs in patients with KD before the initial treatment are compared to the controls. In patients with KD (n = 50), the percentage of EMPs was 1.31 ± 0.16% in KD patients before treatment, which was significantly higher level compared to either healthy children (0.09 ± 0.03%) (n = 25) or children with febrile disease (0.08 ± 0.03%) (n = 25). Boxes and whiskers represent either quartiles or end-points respectively. All values represent the mean ± SD. ****P < 0.0001. (**b**) EMPs are specifically increased in patients with CAL of KD at Stage-2. In patients with NCAL of KD (blue solid circles), EMPs are decreased after defervescence during Stage-1 to Stage-3. In contrast to this, EMPs in patients with CAL of KD (red solid circles) are significantly increased at Stage-2 compared to patients with NCAL of KD, whereas EMPs are comparable between patients with CAL and NCAL of KD either Stage-1 or Stage-3. n = 45 patients with NCAL of KD (blue solid circles), n = 5 patients with CAL of KD (red solid circles). All values represent the mean ± SD. ***P < 0.001. ns, not significant. (**c**) Correlation between EMPs and the maximum Z-score of right or left coronary artery diameters. Significant correlation can be observed between the percentage of EMPs and the maximum Z-score of right or left coronary artery diameters. Blue solid circles show patients with NCAL of KD, and red solid circles indicate patients with CAL of KD.

The percentage of EMPs in the all small particles that were contained in the serum of patients with KD was 1.31 ± 0.16% before initial treatment, which was significantly higher than that of febrile disease controls (0.09 ± 0.03%, p < 0.0001) and healthy controls (0.08 ± 0.03%, *P* < 0.0001) (Fig. 1a). In the KD patients with NCAL, EMPs decreased immediately after defervescence (Stage-2), whereas EMPs increased significantly in the patients with CAL (*P* < 0.01). In both groups of KD, EMPs returned to normal levels within 4 weeks (Stage-3) (Fig. 1b). In addition, there was a statistically significant correlation between the percentage of EMPs and the Z-scores of coronary artery diameters during the acute phase of KD (Fig. 1c).

### MicroRNA array analysis of miRs from patients with acute KD

**Figure 2:**
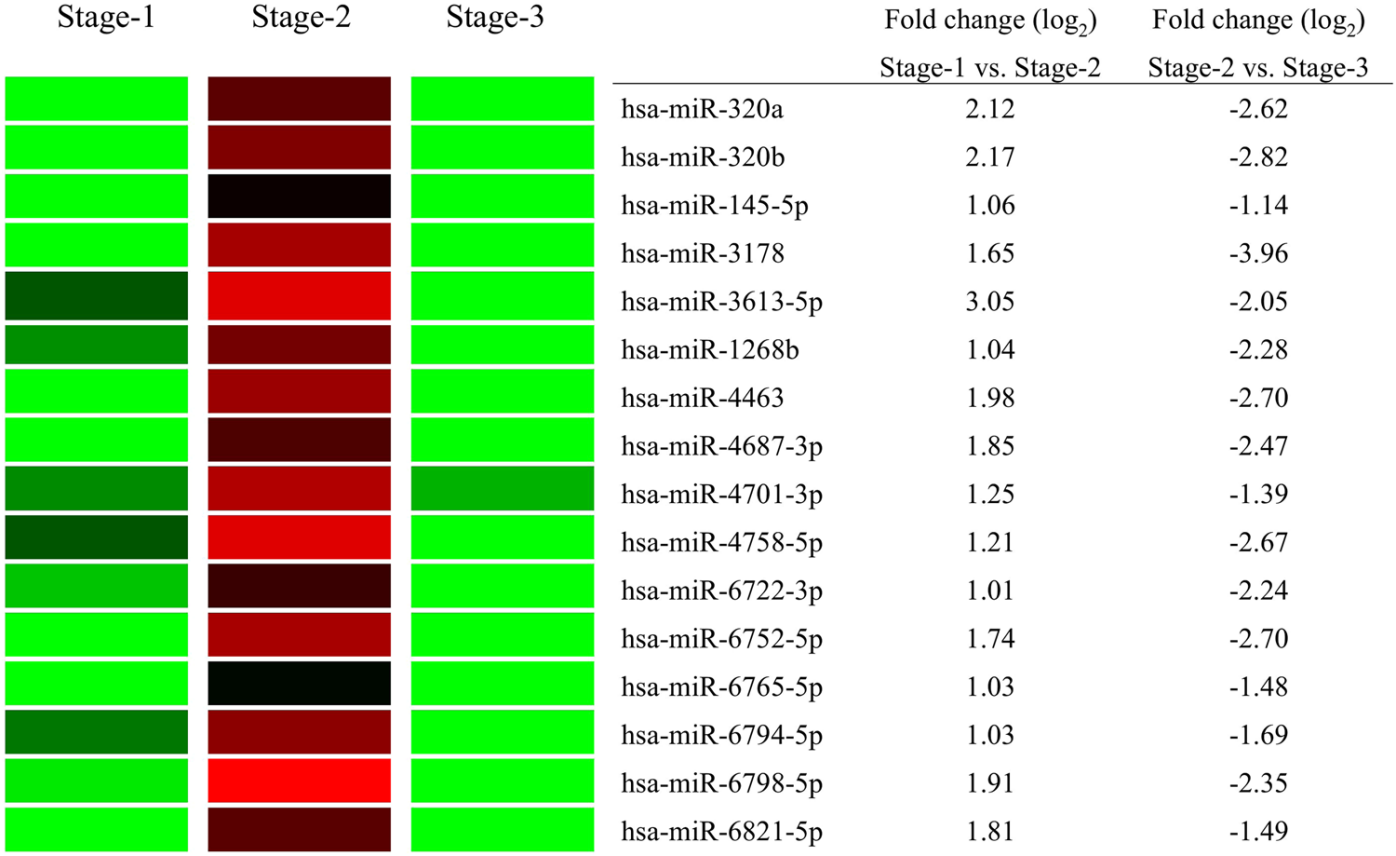
Significantly upregulated 16 miRs are discovered by microRNA array. In patients with CAL of KD, 16 of the 2578 miRs have been screed out as satisfied with both of more than 2 folds (≥1.00 in log2 value) in the Stage-2 compared to the Stage-1 and in each case less than half (≤−1.00 in log2 value) in the Stage-3 compared to the Stage-2.

To elucidate which molecules are associated with adverse outcomes during acute KD, we focused on the role of miRs as the bioactive molecules^7^, and in particular those present in EMPs, which we have shown previously to be involved in the pathogenesis of KD^3^. Generally, circulating MPs are mostly derived from platelets, with a small proportion from endothelial cells^15,16^. We confirmed that the serum samples contained very few CD42b-positive MPs originating from platelet (Supplementary Figure 1), supporting enrichment of EMPs in the serum. In KD patients with CAL, the expression levels of 16 of the 2578 miRs analyzed (0.6%) were significantly upregulated more than 2 folds (≥1.00 in log_2_ value) during the acute phase (Stage-2) compared to the Stage-1 (Fig. 2). Each of these miRs was downregulated more than 50% (≤−1.00 in log_2_ value) during Stage-3 compared to Stage-2 (Fig. 2). In addition, hsa-miR-145-5p, which has previously been reported to be highly expressed in plasma from patients with KD^17^, was elevated at both Stage-1 and Stage-2 (Supplementary Figure 2). Moreover, hsa-miR-145-5p was significantly higher level (1.23 fold) in patients with KD of CAL than that of patients with KD of NCAL at Stage-2 (Supplementary Figure 2). And we confirmed these 16 miRs were higher level in patients with CAL of KD than that of patients with NCAL at Stage-2 (Supplementary Figure 3). These data prompted us to investigate further the role of these 16 miRs.

### Target prediction and pathway enrichment analysis for 16 miRs

**Figure 3:**
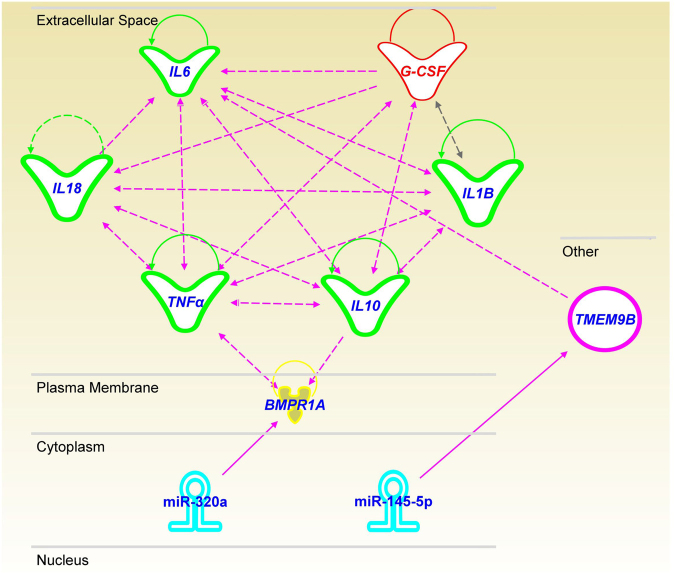
Prediction of target molecules of the selected miRs by *in silico* analysis. The target molecules of selected miRs are predicted using IPA tools. Hsa-miR-320a can interact with *BMPR1A*, and intracellular signaling via BMPR1A may correlate with TNF-α expression. Hsa-miR-145-5p can interact to *TMEM9B*, and then TMEM9B may stimulate IL-6 expression. Thereafter, inflammatory cytokines (TNF-α, IL-1B, IL-6, IL-10 and IL-18) interact each other, leading the upregulation of lesion derived G-CSF level.

We performed in silico analyses of the selected 16 miRs to identify potential mRNA targets. Cutoff values were set as < −1.5 for the mirSVR score of miRanda, >85 for the target score of miRDB, and < −0.3 for total context + score in Target scan to narrow-down the potentially meaningful candidates (less than 300 targets). Only 3 of the 16 miRs, hsa-miR-145-5p, hsa-miR-320a, and hsa-miR-320b, were identified that met these criteria (Tables 3–5). Neutrophil infiltration of the arterial wall of CAL has been reported^18^. For this reason we analyzed the relationships between the miRs identified and genes encoding inflammatory cytokines, tumor necrosis factor-α (*TNF*), interleukin-1β (*IL1B*), interleukin-6 (*IL6*), interleukin-10 (*IL10*) and interleukin-18 (*IL18*), that are commonly secreted from macrophages and genes associated with G-CSF expression^19^, an important chemoattractant protein for the neutrophil infiltration (Fig. 3). IPA analysis showed that hsa-miR-320a can interact with *BMPR1A*, and intracellular signaling via BMPR1A may correlate with TNF-α expression. Moreover, hsa-miR-145-5p can interact with *TMEM9B*, which may stimulate IL-6 expression. The IPA data strongly suggest that these inflammatory cytokines may stimulate each other leading to the up-regulation of G-CSF (Fig. 3).

**Table 3:** Target prediction and pathway enrichment analysis of hsa-miR-145-5p.

**Gene Symbol**	**Gene Description**	**miRANDA**	**miRDB**	**TargetScan**
*ABCE1*	ATP-binding cassette, sub-family E, member 1	−2.33	100	−0.73
*ADD3*	adducin 3	−1.59	98	−0.61
*ATXN2*	ataxin 2	−2	97	−0.54
*DAB2*	disabled homolog 2, mitogen-responsive phosphoprotein	−2.71	100	−0.62
*EPS15*	epidermal growth factor receptor pathway substrate 15	−1.55	87	−0.3
*EXOC8*	exocyst complex component 8	−1.89	93	−0.6
*FSCN1*	fascin homolog 1, actin-bundling protein	−1.97	97	−0.97
*LOX*	lysyl oxidase	−2.24	97	−0.34
*MPZL2*	myelin protein zero-like 2	−2.36	100	−0.61
*NAV3*	neuron navigator 3	−1.99	95	−0.74
*SEMA3A*	sema domain, immunoglobulin domain (Ig), short basic domain, secreted, (semaphorin) 3 A	−1.9	99	−0.65
*ST6GALNAC3*	ST6(alpha-N-acetyl-neuraminyl-2,3-beta-galactosyl-1,3)-N-acetylgalactosaminide alpha-2,6-sialyltransferase 3	−1.87	98	−0.61
*TMEM9B*	TMEM9 domain family, member B	−1.72	85	−0.62
*YTHDF2*	YTH domain family, member 2	−2.3	99	−0.64

*In silico* analysis predicted 14 target mRNAs for hsa-miR-145-5p.

**Table 4:** Target prediction and pathway enrichment analysis of hsa-miR-320a.

**Gene Symbol**	**Gene Description**	**miRANDA**	**miRDB**	**TargetScan**
*BMPR1A*	bone morphogenetic protein receptor, type IA	−1.99	86	−0.4
*CDK13*	cyclin-dependent kinase 13	−1.69	95	−0.46
*DNER*	delta/notch-like EGF repeat containing	−2.23	98	−0.51
*MLLT3*	myeloid/lymphoid or mixed-lineage leukemia; translocated to, 3	−2.28	98	−0.48
*PBX3*	pre-B-cell leukemia homeobox 3	−2.47	100	−0.49
*SEMA3A*	sema domain, immunoglobulin domain (Ig), short basic domain, secreted, (semaphorin) 3A	−1.95	99	−0.39

*In silico* analysis predicted 6 target mRNAs for hsa-miR-320a.

**Table 5:** Target prediction and pathway enrichment analysis of hsa-miR-320b.

**Gene Symbol**	**Gene Description**	**miRANDA**	**miRDB**	**TargetScan**
*LPPR1*	lipid phosphate phosphatase-related protein type 1	−2.71	100	−0.61
*NCAPD3*	non-SMC condensin II complex, subunit D3	−1.9	88	−0.36
*TRIAP1*	TP53 regulated inhibitor of apoptosis 1	−1.79	89	−0.36

*In silico* analysis predicted 3 target mRNAs for hsa-miR-320b.

### MicroRNA ISH

**Figure 4:**
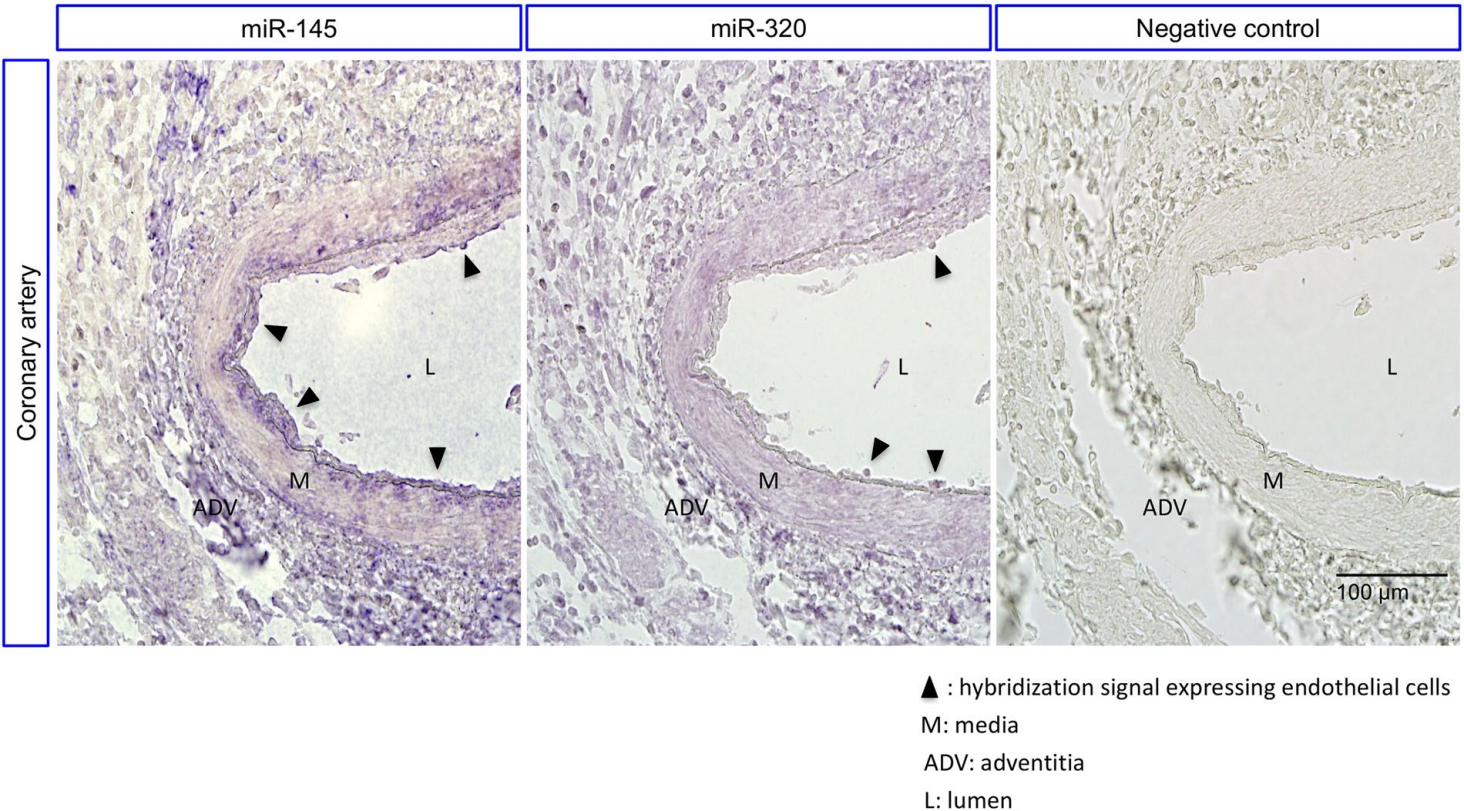
ISH of the selected miRs to sections of coronary artery from a KD patient with CAL. Blue-colored specific hybridization signal indicates expression of the miR-145-5p and miR-320a in endothelial cells of CAL (left and middle), whereas no visible signal can be found on control section (right). This experiment was repeated 3 times. Hsa-miR-145-5p and hsa-miR-320a can be also observed on media and adventitia of CAL. The negative control is a section hybridized without probe. Scale bar = 100 μm.

To demonstrate whether these miRs are expressed in endothelial cells in coronary artery of CAL in Stage-2, we performed microRNA ISH of the 2 miRs (hsa-miR-145-5p and hsa-miR-320a) to sections of coronary artery obtained from a 4-year-old boy with KD, who died when the coronary artery aneurysm ruptured 10 days after diagnosis. Although the expression levels of miRNAs were varied in endothelial cells, specific hybridization signals for hsa-miR-145-5p and hsa-miR-320a could be detected in endothelial cells of the CAL, as well as in the media and adventitia of CAL (Fig. 4). These findings suggest that the miRs may be encapsulated in EMPs, and may appear to be released from endothelial cells to the luminal and abluminal sides, as previously demonstrated^7^. EMPs containing such miRs could be partly transferred to the local tissue resident monocyte/macrophages, and could be partly conveyed to monocytes residing in the bone marrow in these patients.

### Effects of expression of target miRs in THP-1 monocytes

**Figure 5:**
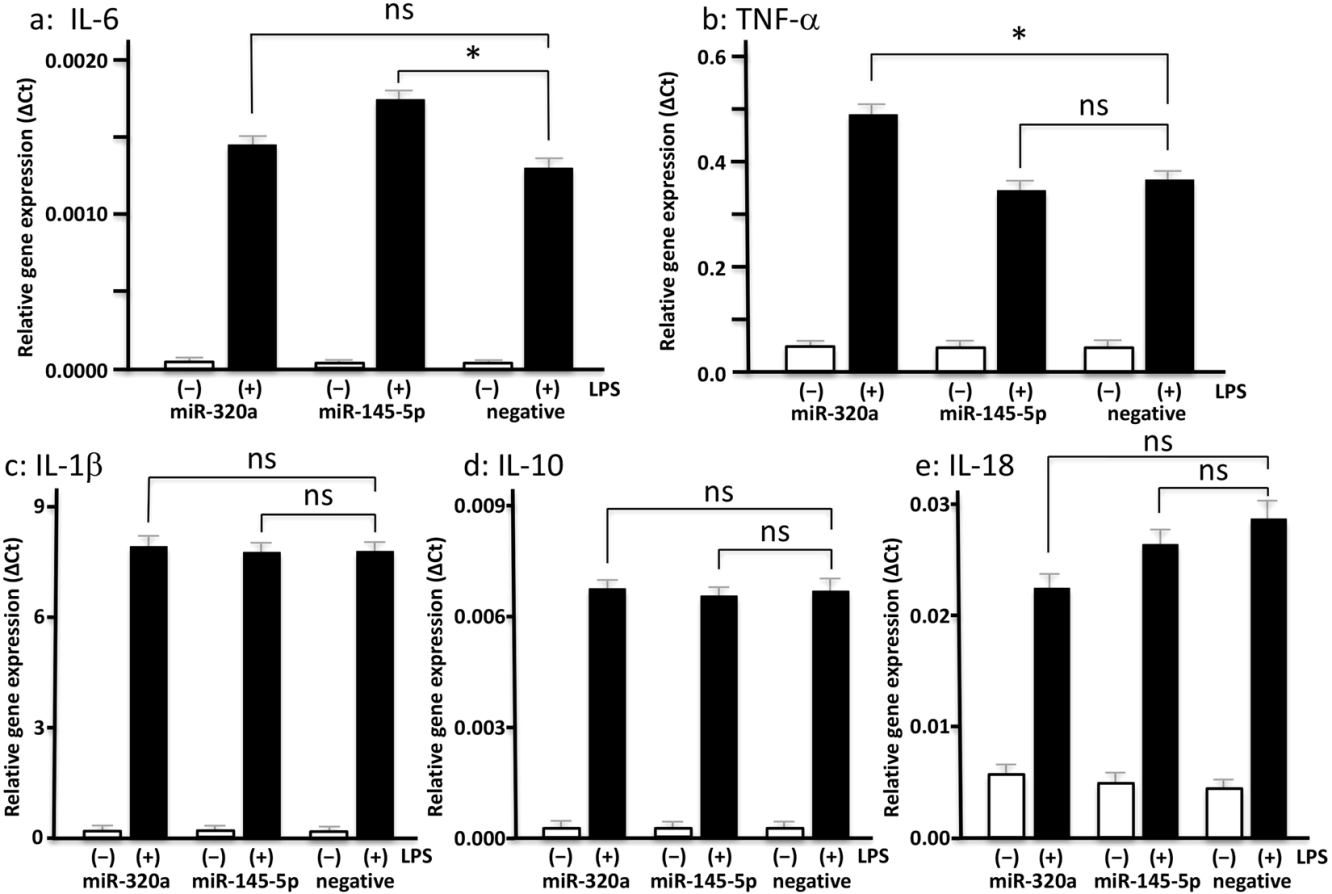
Inflammatory cytokines are upregulated in THP-1 monocytes after transfection of the selected miRs. The induction of inflammatory cytokine mRNAs including *IL6* (**a**), *TNF* (**b**), *IL1B* (**c**), *IL10* (**d**) and *IL18* (**e**) after transfection of hsa-miR-320a, hsa-miR-145-5p and a negative control to THP-1 monocytes in the presence or absence of LPS. This experiment was repeated 3 times. *IL6* is significantly induced by transfection of hsa-miR-145-5p and treated with LPS compared to that of controls (**a**, 32.2% upregulation vs. negative control), whereas transfection of hsa-miR-320a shows similar induction level of *IL6* compared to controls (ns, not significant). *TNF* is significantly induced by transfection of hsa-miR-320a, but not hsa-miR-145-5p (ns), and treated with LPS compared to that of controls (**b**, 24.4% upregulation vs. negative control). Transfection of hsa-miR-145-5p and hsa-miR-320a into THP-1 monocytes resulted in no significant changes in *IL1B* (**c**), *IL10* (**d**) and *IL18* (**e**) mRNA expression in the presence or absence of LPS. All values represent the mean ± SD. *P < 0.05.

**Figure 6:**
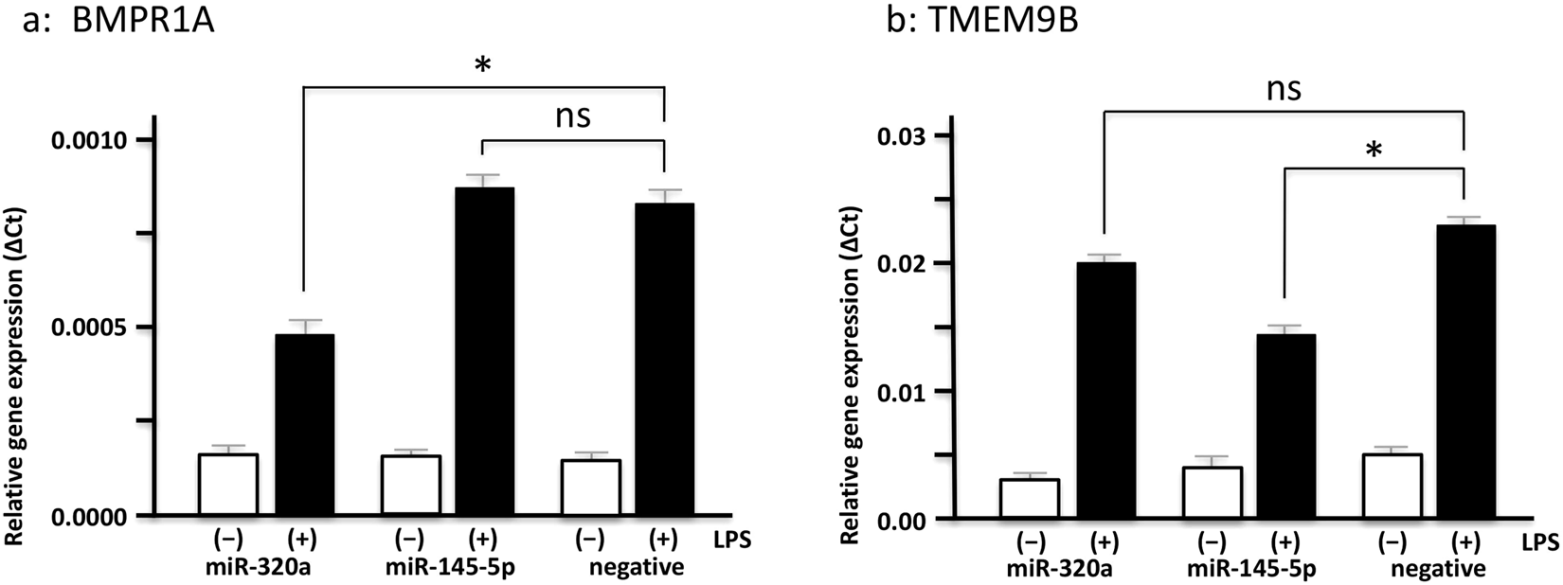
The target mRNAs are downregulated in THP-1 monocytes after transfection of the selected miRs. The *BMPR1A* and *TMEM9B* mRNAs after transfection of hsa-miR-320a, hsa-miR-145-5p and a negative control to THP-1 monocytes in the presence or absence of LPS. This experiment was repeated 3 times. *BMPR1A* is significantly suppressed by transfection of hsa-miR-320a and treated with LPS compared to that of controls (**a**, 30.2% downregulation vs. negative control), whereas transfection of hsa-miR-145-5p shows similar induction level of *BMPR1A* compared to controls (ns, not significant). *TMEM9B* is significantly suppressed by transfection of hsa-miR-145-5p, but not hsa-miR-320a (ns), and treated with LPS compared to that of controls (**b**, 35.0% downregulation vs. negative control). All values represent the mean ± SD. *P < 0.05.

During the inflammatory condition, monocytes/macrophages activated by various stimulants can secrete inflammatory cytokines, such as TNF-α, IL-1β, IL-6, IL-10, and IL-18^20^. To determine whether the 2 miRs encapsulated in EMPs can modulate the expression level of inflammatory cytokines in monocytes/macrophages, we transfected the 2 miRs into the THP-1 monocytes. Previous study reported that hsa-miR-1 could suppress the expression level of *PTK9* mRNA^21^. Validation studies showed that in monocytes transfected with hsa-miR-1, *PTK9* mRNA expression was significantly reduced (87%) at 3 hours post-transfections, and this suppression continued for 24 hours (Supplementary Figure 4). Since LPS efficiently induces inflammation response and is commonly used many experiments for studying the cellular inflammation signaling^7,22–24^, we conducted the experiments in the presence or absence of LPS. Three hours post-transfection cells were harvested and mRNAs of inflammatory cytokines (*TNF*, *IL1B*, *IL6*, *IL10* and *IL18*) and the target mRNAs (*BMPR1A* and *TMEM9B*) were measured by real-time PCR. Significant changes were observed in *IL6* and *TNF* with LPS treatment (Fig. 5a and b). *IL6* expression level was significantly higher in hsa-miR-145-5p and LPS treated cells (p < 0.05), but not in hsa-miR-320a treated cells (Fig. 5a). In contrast, *TNF* expression level was significantly higher in hsa-miR-320a and LPS treated cells (p < 0.05, Fig. 5b), but unchanged in hsa-miR-145-5p treated cells (Fig. 5b). No significant differences in *IL1B*, *IL10*, and *IL18* expression were detected (Fig. 5c–e). Additionally, we have confirmed that *BMPR1A* was significantly suppressed by transfection of hsa-miR-320a and treated with LPS compared to that of controls (a, 30.2% downregulation vs. negative control, p < 0.05), whereas transfection of hsa-miR-145-5p showed similar induction level of *BMPR1A* compared to controls (ns, not significant). *TMEM9B* was significantly suppressed by transfection of hsa-miR-145-5p, but not hsa-miR-320a (ns), and treated with LPS compared to that of controls (b, 35.0% downregulation vs. negative control, p < 0.05) (Fig. 6). Further, the levels of TNF-α in supernatant from hsa-miR-320a transfection experiment were significantly increased (P < 0.05), and the levels of IL-6 in supernatant from hsa-miR-145-5p transfection experiment were significantly increased (P < 0.05), comparted to both of negative microRNA transfection experiments. (Supplementary Figure 5).

## Discussion

Based on our findings, it seems that quantitation of EMPs in serum may serve as a potential biomarker to distinguish patients with acute KD from other febrile diseases. Further, during the acute phase of KD, number of EMPs is significantly increased in patients with CAL compared to patients with NCAL. Strikingly, the percentage of EMPs correlates well with Z-score of coronary artery diameter, suggesting the number of serum EMPs mediates the severity of vascular damage in acute KD. Therefore, EMPs appear to be a sensitive biomarker to predict the severity of vasculitis in acute KD. Several studies have described the origin of microparticles and their association with the disease state in patients with KD^25–27^. Circulating microparticles in blood, derived from endothelial cells and T cells, generally increase after IVIG therapy in patients with KD^25^. Elevation of EMP levels in blood of patients with KD show positive correlations with TNF-α expression, but negatively correlation with albumin expression^26^. These suggest that EMPs may be involved in the development of vasculitis in patients with KD. In addition, EMP levels in plasma have been shown to be increased in patients with acute or subacute KD, and these levels decreased, but remain detectable, during the convalescence stage^27^, suggesting that endothelial damage persists even during KD convalescence. However, few studies have specifically addressed the roles of EMPs in the pathogenesis of vasculitis and the development of CAL in acute KD.

Notably, we identified specific 2 miRs, hsa-miR-145-5p and hsa-miR-320a, in serum enriched for EMPs and depleted of platelet MPs, in patients with KD associated with CAL that may have a role in pathogenesis. Our data derived from *in silico* analyses suggest that hsa-miR-145-5p and hsa-miR-320a regulate expression levels of key target mRNAs associated with KD. Specific miRs involved in the TGF-β pathway have previously been implicated in the pathogenesis of KD, and the generation of myofibroblasts in the arterial wall^17^. It was shown that, during acute phase of KD, hsa-miR-145-5p, which plays a critical role in the differentiation of neutrophils and vascular smooth muscle cells, is expressed at high levels in blood samples from patients with acute KD, but not control subjects^17^. Our findings support these conclusions but also show that hsa-miR-145-5p is expressed in the endothelial cells in KD-associated coronary artery lesions, is detectable in serum from patients with KD, and is able to up-regulate expression of an inflammatory cytokine, IL-6, in THP-1 monocytes by inhibiting *TMEM9B* mRNA. Monocyte/macrophage derived inflammatory cytokines may protract the disease state and worsening of KD^18,28^. Accordingly, hsa-miR-145-5p exerts pleiotropic effects on many type of cells, such as monocytes/macrophages, neutrophils and vascular smooth muscle cells^17^ that contribute to the vasculitis of coronary arteries and forming of CAL in patient with KD.

Previously, it has been reported that hsa-miR-320a is involved in progression of atherosclerosis and endothelial apoptosis^29^; however, a correlation between hsa-miR-320a and progression of KD has not been established. Our findings demonstrate for the first time that hsa-miR-320a is found in the serum of patients with KD, and is able to modulate the expression of the inflammatory cytokine, TNF-α, in THP-1 monocytes by inhibiting *BMPR1A* mRNA. It was previously suggested that TNF-α induced cell status alteration might intimately correlate intracellular signaling via BMPR1A^30^. Accordingly, hsa-miR-320a function to modulate TNF-α production mediated by BMPR1A signaling. Therefore, from the above data, hsa-miR-320a may participate in the regulation of the expression of inflammatory cytokines in collaboration with hsa-miR-145-5p, and may advance the vasculitis of coronary arteries resulting in CAL in patients with KD.

We showed our data previously reported miRs about KD such as miR-125a^31^, miR-93^11^, and miR-483^32^. In Stage-2 with CAL, miR-125a (Fold change 1.26) and miR-93 (Fold change 1.10) were upregulated compared with fever control, respectively. In contrast to these, miR-483 was downregulated (Fold change 0.60). Apoptosis of endothelial cells was induced by a decrease of MMK7 which is one of the targets of miR-125a. VEGF-A expression was suppressed by miR-93. Downregulation of miR-483 was appeared to alter the expression of connective tissue growth factor (CTGF). These alteration of the targets of KD related miRs likely influences vascular wall injury and aneurysm formation in acute KD.

The primary histopathological features of coronary arteries during the acute phase of KD include swelling of endothelial cells and subendothelial edema following initial neutrophil extravasation and infiltration in the lesion. Neutrophils induce endothelial damage through the production of oxygen radicals, and secretion of elastase and MMP-9 during the acute phase of KD^33,34^. Pathologically, necrosis of the coronary artery wall results in deposition of fibrin and cellular debris, and infiltration of neutrophils^18,28^. G-CSF has been considered the key chemoattractant molecule for the neutrophil recruitment to the lesion. Serum levels of G-CSF typically increase during the acute phase of KD^35^. G-CSF specifically and markedly expands hematopoietic stem/progenitor cells followed by mobilization of neutrophils from the bone marrow, and accelerates the functional activities of neutrophils^35^. In addition, G-CSF serum levels are significantly higher in patients with CAL of KD than patients with NCAL either before or after IVIG treatment^36^. It has been reported that elevated numbers of circulating neutrophils are involved in the development of CAL^37^. Therefore it is probable that G-CSF plays an important roles in the pathogenesis and progression of CAL. Further studies are needed to examine the relationships between the kinetics of cytokines expression and contribution of neutrophils to the formation of CAL in the pathogenesis of KD during the acute phase.

We conclude that specific miRs encapsulated in EMPs may modulate the secretion of inflammatory cytokines from monocytes/macrophages. Such inflammatory related cells, and other cells consisting of the coronary artery to be able to induce G-CSF in responsible to monocyte/macrophage derived inflammatory cytokines^38–40^. The cellular/molecular inflammatory cascade activated in the lesion allows neutrophils originating in the bone marrow to infiltrate CAL. Therefore, in order to prevent the progression of CAL in the patients with KD, hsa-miR-145-5p and hsa-miR-320a, which express in endothelial cells in CAL and may be encapsulated in EMPs, might be novel therapeutic targets for the prevention and treatment of KD. We suggest that further efforts are needed to develop the highly efficient molecular targeting therapies for KD.

### Study limitations

We used THP-1 monocytes as a target in the gene expression experiments analyzing the effects of miRs. However, it will be necessary to confirm these data using other cell types, such as endothelial cells, vascular smooth muscle cells, and white blood cells, because EMPs may act on various cell types. Because of the limitation of sample volume from patients, and limitation of completeness and accuracy of flow cytometry, we used EMP enriched and platelet MP reduced serum samples for extracting miRs. Technical advances resulting in the isolation of specific MP fractions may improve the specificity of these findings.

Another limitation is that only 5 patients with CAL were enrolled in this study. However, since only 10% of KD patients develop CAL after 1 month of disease onset^2^, this is the expected number given that 50 patients were enrolled in the study.

Finally, we didn’t directly prove that the BMPR1A suppressed the *TNF* mRNA and the TMEM9B suppressed the *IL-6* mRNA; however, we clearly showed that transfection of hsa-miR-145-5p and hsa-miR-320a suppressed *TMEM9B* and *BMPR1A* mRNAs, and increased IL-6 and TNF-α proteins in culture supernatants, respectively (Fig. 6 and Supplementary Figure 5). Previous reports supportively indicated that TMEM9B significantly regulated IL-6 in human dermal fibroblasts^41^, and BMPR1A suppressed the expression of TNF-α in macrophage cell line^42^. Accordingly, our results of transfection studies might be trustworthy.

## Conclusions

These results suggested that EMPs could serve as a sensitive marker for characterizing the severity of endothelial damage and vasculitis in acute KD. Moreover, these specific miRs, hsa-miR-145-5p and hsa-miR-320a, may participate in modulation of inflammatory cytokine expression levels, and may contribute to pathogenesis of vasculitis in acute KD. Our novel findings might offer a new clinical approach for the treatment of KD. Further efforts are needed to develop the highly efficient molecular targeting therapies for KD.

## Methods

### Enrollment of patients

Fifty patients with KD (aged 4 months to 14 years, 30 males, 20 females), and 50 controls (25 non-KD febrile and 25 healthy children) were enrolled from April 2013 to March 2016 in this multicenter clinical trial regulated in Toyama University Hospital and related facilities (Table 1). The study was approved by the Ethics Committee of the University of Toyama and performed in accordance with the Declaration of Helsinki: Patients and controls were enrolled after informed consent.

Patients with KD who responded to IVIG treatment and alleviated fever within 48 hours were designated as responders, while patients with KD who did not respond to IVIG were designated refractory patients. Further, patients with KD in the convalescent phase were divided into two subgroups; those with coronary artery lesions (CAL, coronary artery diameter Z-score > 2.5, n = 5) or no coronary artery lesion (NCAL, Z-score ≦ 2.5, n = 45) (Table 2). Blood samples were collected at three time points; Stage-1, at the time of diagnosis before IVIG treatment; Stage-2, immediately after IVIG infusion; and Stage-3, at 2-4 weeks after the onset of the disease.

### Flow cytometry of EMPs

Serum samples were fractionated by centrifugation at 3,000 g for 10 minutes, and then coarse particles and cellular debris were removed by centrifugation at 12,000 g for one minute. Small sized particles in the fractionated serum samples were used for further analysis. Fifty μl of the fractionated serum samples were incubated with 5 μl FITC-conjugated mouse anti-human CD144 (BD Pharmingen, San Diego, CA) and 5 μl PE-conjugated mouse anti-human CD42b (BD Pharmingen) for 20 minutes on ice. Labeled EMPs were measured by Cytomics FC500 (Beckman Coulter, Fullerton, CA). EMPs were gated between 100 nm to 1,000 nm in diameter, corresponding to the size of the EMPs, and the percentage of CD144 positive MPs among all the particles in the gated range were calculated.

### RNA extraction

Total RNA in fractionated serum samples (400 μl) was extracted using miRNeasy Serum/Plasma Kits (QIAGEN GmbH, Hilden, Germany), according to the manufacturer’s instructions, and quantitated using a NanoVue (GE imagination at work). The extracted RNA samples were stored at −150 °C until further processing.

### MiRNA microarray

MiRNA expression profiling was performed on 130 ng of total RNA using a miRNA 4.0 microarray (Affymetrix^®^ GeneChip^®^), according to the manufacturer’s instructions. The microarray covers 2,578 human miRNA probes.

### Analysis of the result of microarray data

In order to identify of the specific miRNAs that were differentially expressed between patients with CAL of KD and those of NCAL or controls, the method of quantitative log_2_ metrics was used for the data analysis. Greater than 1.0 (higher-expression) or less than −1.0 (lower-expression) fold changes, using the log_2_ scale, were considered to be biologically significant^43^.

### Target prediction for miRNAs

Publicly accessible algorithms, including TargetScan, miRanda, and miRDB, were used for *in silico* analysis and identification of miRNA targets. The data were also analyzed using Ingenuity Pathways Analysis (IPA) tools (Ingenuity Systems, Mountain View, CA), a web-based application that enables the discovery, visualization and exploration of molecular interaction networks using miRNA expression data.

### ***In situ*hybridization (ISH) of miRNAs**

ISH was performed using formalin-fixed and paraffin-embedded sections from a coronary artery lesion obtained from a patient during the acute phase of KD. Double-Dig-labeled probes of the hsa-miR-145-5p, and hsa-miR-320a were obtained from Exiqon (miRCURY LNA^TM^ Detection probe; Vedbaek, Denmark), and was used according to the manufacturer’s instructions. The images were obtained using a BX51 microscopy system (Olympus, Tokyo, Japan).

### Cell culture and stimulation experiments

The THP-1 monocyte cells (National Institutes of Biomedical Innovation, Health and Nutrition, Japan) were cultured using RPMI-1640 containing 10% heat-inactivated fetal calf serum (FCS), 1% penicillin G and 0.1% gentamicin, at 37 °C in a humidified atmosphere containing 5% CO_2_. A total of 500,000 THP-1 cells were seeded in each well of a 6-well plate containing 2 ml of fresh culture medium. For the validation study, after 24 hours the cells were transfected with 25 pmol of a control miR, hsa-miR-1 (50 μM final concentration: miScript miRNA Mimic, Life Technologies, Carlsbad, CA) using 7.5 μl Lipofectamine^®^ RNAiMAX Transfection Reagent (Life Technologies) according to the manufacturer’s instructions. THP-1 cells were harvested at 0, 3, 6, and 24 hours after transfection, and total RNA was extracted by an RNeasy Mini Kit (QIAGEN GmbH, Hilden, Germany). The expression level of an mRNA coded protein tyrosine kinase 9 (*PTK9*), a target of hsa-miR-1, was quantitated by real-time PCR. Once the optimum transfection time was established, cells were similarly transfected with hsa-miR-145-5p, hsa-miR-320a and a negative control miRNA Mimics, in the presence or absence of the 0.2 μM lipopolysaccharide (LPS). The cells were harvested on 3 hours post transfection and total RNA was isolated. mRNAs encoding tumor necrosis factor-α (*TNF*), interleukin-1β (*IL1B*), interleukin-6 (*IL6*), interleukin-10 (*IL10*), interleukin-18 (*IL18*), bone morphogenetic protein receptor type 1A (*BMPR1A*) and TMEM9 domain family member B (*TMEM9B*) were quantitated by real-time PCR. All experiments were performed in triplicate and RT-PCR primers are shown in Table 6.

**Table 6:** PCR primers sequences (5′ to 3′).

Primers	Sequences (forward)	Sequences (reverse)
GAPDH	atgttcgtcatgggtgtgaa	atgttcgtcatgggtgtgaa
IL-1B	gggcctcaaggaaaagaatc	ttctgcttgagaggtgctga
IL-6	aggagacttgcctggtgaaa	caggggtggttattgcatct
IL-10	tgccttcagcagagtgaaga	ggtcttggttctcagcttgg
IL-18	gcaccccggaccatattta	tcctgggacacttctctgaaa
TNF-α	cagagggcctgtacctcatc	ggaagacccctcccagatag
PTK9	cccaaggattcagctcgtta	gcagtcaactcatccccatt
BMPR1A	gagttgctgcattgctgacc	tcttccacgatccctcctgt
TMEM9B	ggggcctgatgtagaagcat	caaaaggctggtgatcccca

### Analysis on cell culture supernatant level of TNF-α and IL-6 by ELISA

THP-1 transfection experiment’s supernatant concentrations of TNF-α and IL-6 were determined by a sandwich enzyme-linked immunosorbent assay (ELISA) using commercial kits (Quantikeine ELISA, R&D Systems, USA & Canada), according to the manufacturer’s instructions.

### Statistics

Statistical significance was determined using Student’s t-test, or one- or two-way analysis of variance (ANOVA) with Tukey’s multiple comparisons as a post-hoc analysis for ANOVA. *P* values less than 0.05 were considered statistically significant. Graphs were drawn using GraphPad Prism 6.04 (GraphPad Software, Inc., La Jolla, CA). Quantified data are presented as mean ± SD.

## Electronic supplementary material

Supplementary Information
