# Supplementary material for: MicroRNA-145-5p and microRNA-320a encapsulated in endothelial microparticles contribute to the progression of vasculitis in acute Kawasaki Disease
Source: Sci Rep. 2018 Jan 17;8:1016. doi: 10.1038/s41598-018-19310-4 (PMC5772486; doi:10.1038/s41598-018-19310-4)
Supplement: Supplementary file 1 — Supplementary Information [file 41598_2018_19310_MOESM1_ESM.pdf]

SUPPLEMENTARY INFORMATION

MicroRNA-145-5p and microRNA-320a encapsulated in endothelial microparticles contribute to the progression of vasculitis in acute Kawasaki Disease.

Hideyuki Nakaoka, MD, PhD[1], Keiichi Hirono, MD, PhD[1], Seiji Yamamoto, PhD[2], Ichiro Takasaki, PhD[3], Kei Takahashi, MD, PhD[4], Koshi Kinoshita, PhD[5], Asami Takasaki, MD, PhD[1], Naonori Nishida, MD, PhD [1], Mako Okabe, MD[1], Wang Ce, MD[1], Nariaki Miyao, MD[1], Kazuyoshi Saito, MD, PhD[1], Keijiro Ibuki, MD, PhD[1], Sayaka Ozawa, MD, PhD[1], Yuichi Adachi, MD, PhD[1], Fukiko Ichida, MD, PhD[1]

1) Department of Pediatrics, Faculty of Medicine, University of Toyama, Toyama, Japan

2) Department of Pathology, Faculty of Medicine, University of Toyama, Toyama, Japan

3) Division of Molecular Genetics Research, Life Science Research Center, University of Toyama, Toyama, Japan

4) Department of Pathology, Toho University Ohashi Medical Center, Tokyo, Japan

5) Department of Legal Medicine, Faculty of Medicine, University of Toyama, Toyama, Japan

Address for corresponding author:

Fukiko Ichida, MD, PhD

Department of Pediatrics, Faculty of Medicine, University of Toyama

2630 Sugitani, Toyama, Toyama, 930-0194 Japan

Tel: +81-76-434-7311

Fax: +81-76-434-5029

E-mail address: fkichida@ctg.u-toyama.ac.jp

Supplementary Figure 1: Flow cytometry analysis of CD42b positive platelet derived MPs.

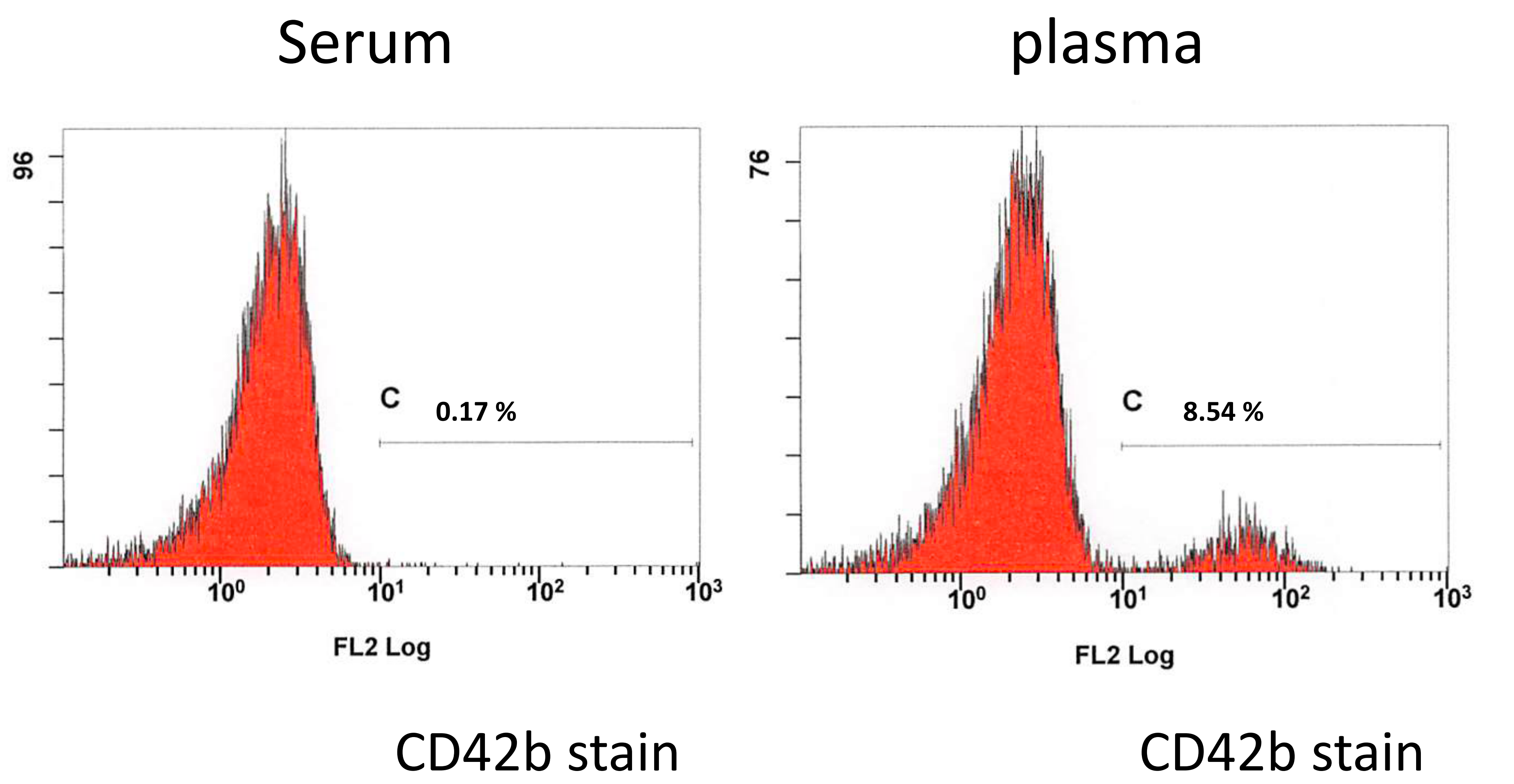

Whereas CD42b positive platelet derived MPs are detectable level in plasma,
very few CD42b positive platelet derived MPs are measured in serum by flow cytometry.

Supplementary Figure 2: Strongly upregulated hsa-miR-145-5p is observed in patients with KD of CAL during Stage-1 to Stage-3.

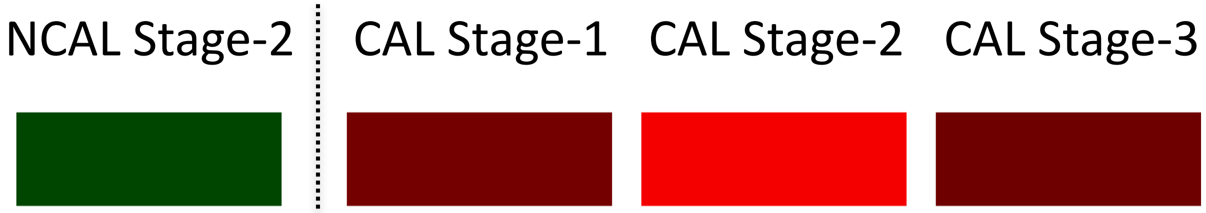

	Fold change (log_2)<br>Stage2;<br>NCAL vs. CAL
hsa-miR-145-5p	1.23

Hsa-miR-145-5p is expressed more highly during Stage-1, and continued to be highly expressed during Stage-2 and Stage-3 in patients with KD of CAL. Worthy of note, hsa-miR-145-5p expression level is significantly higher in patients with KD of CAL than that of patients with KD of NCAL during Stage-2.

Supplementary Figure 3: The specific 16 miRs with Stage-2 KD patient samples of CAL and NCAL

Stage2; NCAL vs. CAL	Fold change
hsa-miR-320a	1.59
hsa-miR-320b	1.74
hsa-miR-145-5p	2.35
hsa-miR-3178	1.36
hsa-miR-3613-5p	1.99
hsa-miR-1268b	1.57
hsa-miR-4463	1.65
hsa-miR-4687-3p	1.31
hsa-miR-4701-3p	2.24
hsa-miR-4758-5p	1.34
hsa-miR-6722-3p	1.48
hsa-miR-6752-5p	1.70
hsa-miR-6765-5p	1.59
hsa-miR-6794-5p	1.28
hsa-miR-6798-5p	1.58
hsa-miR-6821-5p	1.18

These 16 miRs were higher level in patients with CAL of KD than that of patients with NCAL of KD at Stage-2.

Supplementary Figure 4: The temporal change in *PTK9* mRNA expression after transfection of THP-1 monocytes with hsa-miR-1.

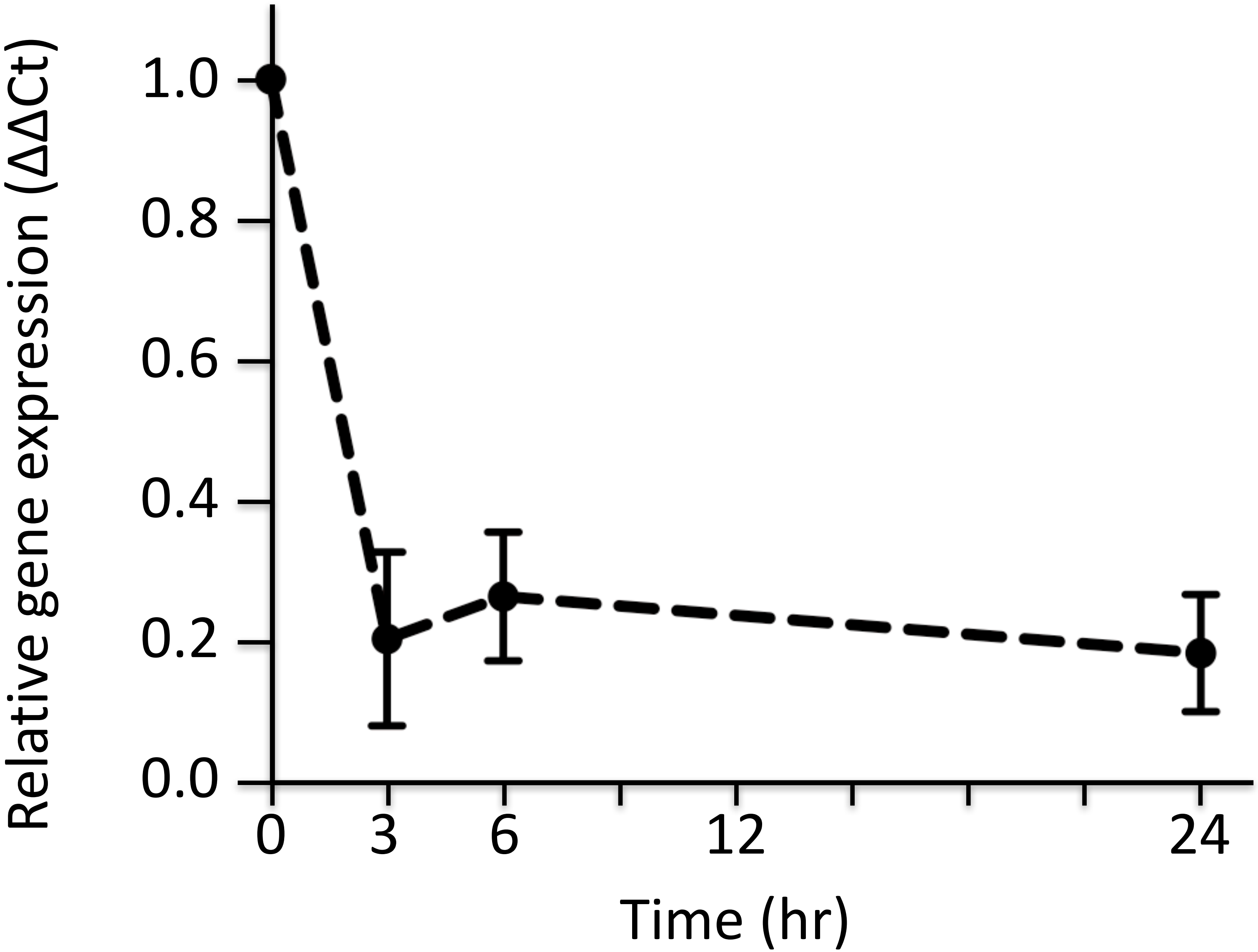

Supplementary Figure 5: The sequential change of expression of TNF-α and IL-6

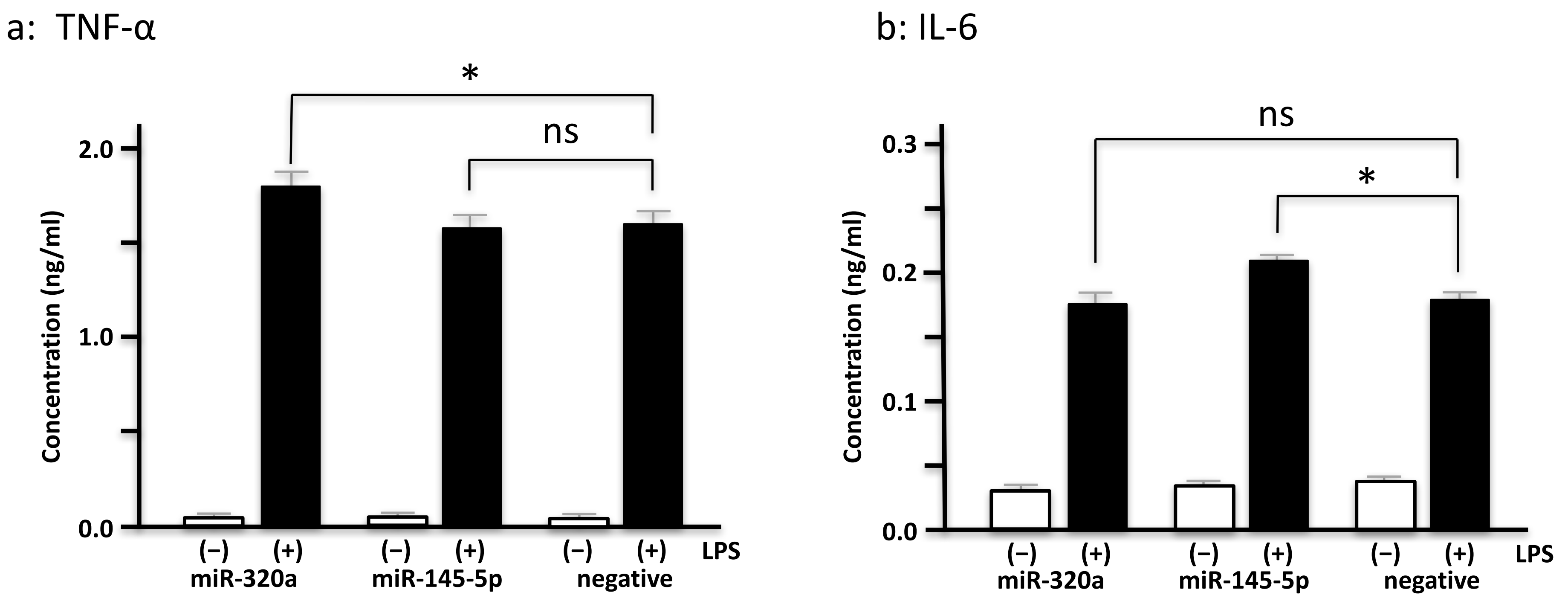

The levels of TNF-α in supernatant from hsa-miR-320a transfection experiment were significantly increased ($P<0.05$), and the levels of IL-6 in supernatant from hsa-miR-145-5p transfection experiment were significantly increased ($P<0.05$), comparted to both of negative microRNA transfection experiments.
